# IL-10 Is Significantly Involved in HSP70-Regulation of Experimental Subretinal Fibrosis

**DOI:** 10.1371/journal.pone.0080288

**Published:** 2013-12-20

**Authors:** Yang Yang, Atsunobu Takeda, Takeru Yoshimura, Yuji Oshima, Koh-Hei Sonoda, Tatsuro Ishibashi

**Affiliations:** 1 Department of Ophthalmology, Graduate School of Medical Sciences, Kyushu university, Fukuoka, Japan; 2 Department of Ophthalmology, Yamaguchi University School of Medicine, Ube, Yamaguchi, Japan; Charite Universitätsmedizin Berlin, Germany

## Abstract

Subretinal fibrosis is directly related to severe visual loss, especially if occurs in the macula, and is frequently observed in advanced age-related macular degeneration and other refractory eye disorders such as diabetic retinopathy and uveitis. In this study, we analyzed the immunosuppressive mechanism of subretinal fibrosis using the novel animal model recently demonstrated. Both TLR2 and TLR4 deficient mice showed significant enlargement of subretinal fibrotic area as compared with wild-type mice. A single intraocular administration of heat shock protein 70 (HSP70), which is an endogenous ligand for TLR2 and TLR4, inhibited subretinal fibrosis in wild-type mice but not in TLR2 and TLR4-deficient mice. Additionally, HSP70 induced IL-10 production in eyes from wild-type mice but was impaired in both TLR2- and TLR4-deficient mice, indicating that HSP70-TLR2/TLR4 axis plays an immunomodulatory role in subretinal fibrosis. Thus, these results suggest that HSP70-TLR2/TLR4 axis is a new therapeutic target for subretinal fibrosis due to prognostic CNV.

## Introduction

Age-related macular degeneration (AMD) is the leading cause of irreversible blindness, which is estimated to affect more than 8 million individuals in the USA, and the advanced form of the disease affects more than 1.75 million individuals [1]. The neovascular form of the disease is characterized by the invasion of new pathological vessels under the macula (choroidal neovascularization, CNV) and it is associated with a rapid and severe decrease of vision. Numerous studies about the mechanism of CNV formation have been reported, many of which resulted in the initiation of clinical trials. The accumulated knowledge has led to the development of several therapeutic strategies for AMD, such as verteporfin photodynamic therapy (PDT), anti-vascular endothelial growth factor (VEGF) therapy, and combined therapy [2]. In contrast, little is known regarding the molecular mechanism(s) of tissue scar formation in CNV. Since fibrotic changes in the foveal CNV lesion frequently result in severe, permanent visual impairment in patients with wet AMD, the treatment of tissue fibrosis in the late stage of AMD is of great interest.

Fibrosis is a common pathophysiological response of many tissues to chronic injury, which can be considered wound repair, mostly associated with robust inflammatory response [3]. Recruitment of inflammatory cells and the subsequent laying down of extracellular matrix during wound repair is a healthy response to tissue damage. However, this evolutionary adaptation comes at the cost of an excessive and poorly ordered matrix deposition and fibrosis, which affects normal tissue architecture and ultimately can disable proper functioning of tissues.

Toll-like receptors (TLRs) are germline-encoded pattern recognition receptors that are important in the innate immune system involved in initial step of host defense against microorganisms. Accumulated lines of evidence indicate that TLRs are also activated by endogenous ligands such as high mobility group box 1 (HMGB1), hyaluronan, and heat shock proteins released from damaged tissues, termed damaged associated molecular patterns (DAMPs). Such innate immune responses contribute not only to inflammation, but also to physiological and pathological repair processes including fibrosis.

HSPs are a family of highly conserved proteins found in all eukaryotes and prokaryotes. The HSP70 family, located in the cytosol and the nucleus of various kinds of cells, is released in response to cellular stress such as UV light, trace metals, and xenobitics. Several studies have shown that extracellular HSPs have important immunomodulatory functions [4]. Induction of HSP70 is protective in animal models of various diseases, such as inflammatory bowel diseases [5], ultraviolet light-induced skin damage [6] and Alzheimer’s disease [7]. Furthermore, several studies have reported that inflammation, fibrosis and dysfunction are suppressed in transgenic mice expressing HSP70 [8]. HSP70 specifically binds both TLR2 and 4 [9]. Dybdahl et al. reported that autologous release of HSP70 after open heart surgery induces a proinflammatory response in innate immune cells potentially mediated via TLR2 and TLR4 [1], [10].

In the present study, we investigated the involvement of the HSP70 and its downstream TLR2 and TLR4 signalings in the formation of subretinal fibrosis with animal model which we recently introduced. Moreover, the mechanisms underlining the HSP70-regulation of on subretinal fibrosis were examined. Here we show that exogenous HSP70, by inducing anti-inflammatory cytokine IL-10, ameliorates experimental subretinal fibrosis formation through both TLR2- and TLR4-dependent mechanisms.

## Materials and Methods

### Ethics Statement

This study was carried out in strict accordance with the ARVO Statement for the Use of Animals in Ophthalmic and Vision Research. The protocol was approved by the Committee on the Ethics of Animal Experiments of Kyushu University (Permit Number: A21-147-1, A23-088-0 and A23-220-0). All surgery was performed under sodium pentobarbital anesthesia, and all efforts were made to minimize suffering.

### Mice

C56BL/6 (WT) mice were obtained from SLC Japan (Shizuoka, Japan). TLR2-deficient (TLR2KO) and TLR4-deficient (TLR4KO) mice were obtained from WPI Immunology Frontier Research Center, Osaka University and backcrossed to WT mice for more than 10 times. All animals were housed in specific pathogen free conditions at Kyushu University and treated humanely. Experiments conformed to the ARVO Statement for the Use of Animals in Ophthalmic and Vision Research.

### Preparation of PECs

Macrophage-rich (peritoneal exudate cells) PECs were obtained from WT, TLRKO and TLR4KO mice that received 2.5 ml of 3% aged thioglycolate solution (Difco, Detroit, Ml) 3 days before sacrifice, plated in 10 cm dish, from which nonadherent cells were washed out with PBS. The remaining adherent cells were incubated with 0.02% EDTA and collected by vigorous pipetting. Cells were counted and resuspended with PBS.

### Subretinal Fibrotic Model

Subretinal fibrotic models were generated as previously described [11]. Briefly, laser photocoagulation (wave length 532 nm, 0.1 s, spot size 75 um, power 200 mW) was performed to the retina to make subretinal bubble and rupture Bruch membrane in WT, TLR2 KO, or TLR4 KO mice. PECs (4×10^4^) collected as above without any stimulation were inoculated into the subretinal space. On day 7 after PEC-inoculation, mice were sacrificed and eyes were enucleated, which were then fixed in 4% paraformaldehyde. Choroidal flatmounts were prepared and stained with anti-glial fibriary acidic protein (GFAP) antibody conjugated with FITC.

### Vitreous Cavity Injection

1 ng of anti-TLR2 or TLR4 neutralizing antibodies or PBS was injected into vitreous cavity with subretinal inoculation of PECs in WT mice. 2 hours after PEC inoculation, 50 ng (25 µg/ml, 2 µl) of recombinant human heat shock protein 70 (HSP70) (Assay designs, # NSP-555) or control PBS was administered into vitreous cavity in WT or TLR4 KO mice. 2 hours after PECs-inoculation, 1 µg of lipopolysaccharide (LPS) or PBS was administered into vitreous cavity in WT mice.

### Quantitative Real-time Reverse Transcriptase (RT)-PCR

0, 24, 48 or 72 h after PEC inoculation, eyes were enucleated under deep anesthesia, the conjunctival tissue was removed, and the remaining eye tissues (cornea, iris, vitreous body, retina, choroids and sclera) were homogenized using a Biomasher (Nippi Inc., Tokyo, Japan). Then homogenized tissues were immersed in TRIzol reagent (Invitrogen Corp., Carlsbad, CA) and processed for RNA isolation. The reverse-transcriptase cDNAs were then subjected to real-time PCR using SYBR Premix Ex Taq (Takara Bio Inc., Otsu, Japan) and a Light Cycler (Roche Diagnostics GmbH, Mannheim, Germany). The primers were as follows; 5′-AACTGCACCCACTTCCCAGTC-3′ and 5′-CATTAAGGAGTCGGTTAGCAG3′for IL-10, 5′-GGCTGATCGGCCGCAAGTT-3′ and 5′-AACTGCACCCACTTCCCAGTC-3′ for HSP70, 5′-GATGACCCAGATCATGTTTGA-3′ and 5′-GGAGAGCATAGCCCTCGTAG-3′ for beta-actin. All estimated mRNA values were normalized to beta-actin mRNA levels. Each experiment was carried out at least twice and representative data are shown.

### RPE and PEC Cell Culture

Retinal pigment epithelial (RPE) cells were prepared from eyes of WT, TLR4KO and TLR2KO mice as previously described, [2], [12] and then incubated until almost confluent in DMEM supplemented with 20% heat-inactivated fetal calf serum, 100 U/ml penicillin, 100 µg/ml streptomycin, 1% L-glutamine, and 0.1 mM non-essential amino acids at 37°C in 5% CO_2_. RPE cells or PECs were plated in six-well dishes (Collagen-Coated Microplate 6 Well with Lid Collagen Typel, IWAKI, Chiba, Japan), and then the media was replaced to 1 ml of media. Cells were stimulated at doses of 0, 0.1, 0.3 and 1 ng/ml recombinant human Hsp70 for 48 h. RPE cells were stimulated with 0, 0.1,1.0 and 10 µg/ml LPS for 48 h.

### Cytokine ELISA

Following stimulation of primary RPE cells and PECs by recombinant human Hsp70 (0, 0.1, 0.3 and 1 ng/ml) or LPS (0, 0.1, 1.0 and 10 µg/ml) for 48 h, supernatants were harvested. IL-10 levels were measured by enzyme-linked immunosorbent assay (ELISA) following the manufacturer’s instructions (eBioscience, Cat# 88-7104-88).

For protein analysis, the retina-RPE-choroid complex were isolated from eyes of WT mice 48 hours after PBS or HSP70 inoculation, pooled, lysed, homogenized, centrifuged, then assessed by ELISA. The total protein concentration was determined with Bio-Rad protein assay reagent kit (Bio-Rad Laboratories, Inc, Hercules, CA).

### Immunohistochemistry

72 h after PEC inoculation following intravitreal injection of recombinant HSP70 or control PBS, eyes were enucleated and immediately frozen (−80°C) in tetrafluorethane overnight. Then 6 µm transverse sections were cut on a Leica Microtome (Leica Cryostat model CM 1800, Germany) and placed on silanised glass slides. Sections were stained with hematoxylin-eosin (HE) for general morphology. Dehydrated sections were blocked with H_2_O_2_ (0.3% in methanol) and skim milk (2% in PBS) for 30 minutes, respectively. Then sections were incubated overnight with primary antibodies (Rabbit anti-mouse IgG, 1∶200; and TLR4, 1∶50; and TLR2, 1∶200; and HSP70, 1∶50) at 4°C in moist chambers, followed by incubation with the secondary antibody (Goat anti-rabbit IgG, 1∶200) for 30 minutes at room temperature. Finally, the signals were detected using the fluorescein streptavidin (1∶50). The reaction was allowed to develop for approximately 10 minutes and staining was halted by washing in PBS/Tween20 3 times for 5 minutes each.

Primary RPE cells from mice were treated with recombinant human HSP70 (1 ng/ml) for 48 h. PECs were added to the primary culture of RPEs some samples in the absence or presence of HSP70 (1 ng/ml), IL-10 (10 ng/ml). Cells were washed with PBS 5 times, fixed with cold methanol (1∶1) for 1 min and blocked with 20% Blocking One (Nacalai Tesque, #03953-95) for 30 minutes, then incubated with the following primary antibodies (Rabbit anti-mouse IgG, 1∶200; and TLR4, 1∶50; and TLR2, 1∶200; and HSP70, 1∶50) at 4°C overnight, followed by incubation with the secondary antibody (Goat anti-rabbit IgG, 1∶200) for 60 minutes at room temperature. The nuclei of cells were counterstained with Hoechst 33342 (1∶400, Molecular Probes) for 10 minutes.

### Statistics

Data were analyzed for significant differences between experimental groups with either ANOVA/Scheffe’s test (more than three groups) or Student’s t-test (two groups). P values<0.05 were considered significant.

## Results

### Augmented Subretinal Fibrosis by Blockade of TLR2 and TLR4 Signaling

**Figure 1:**
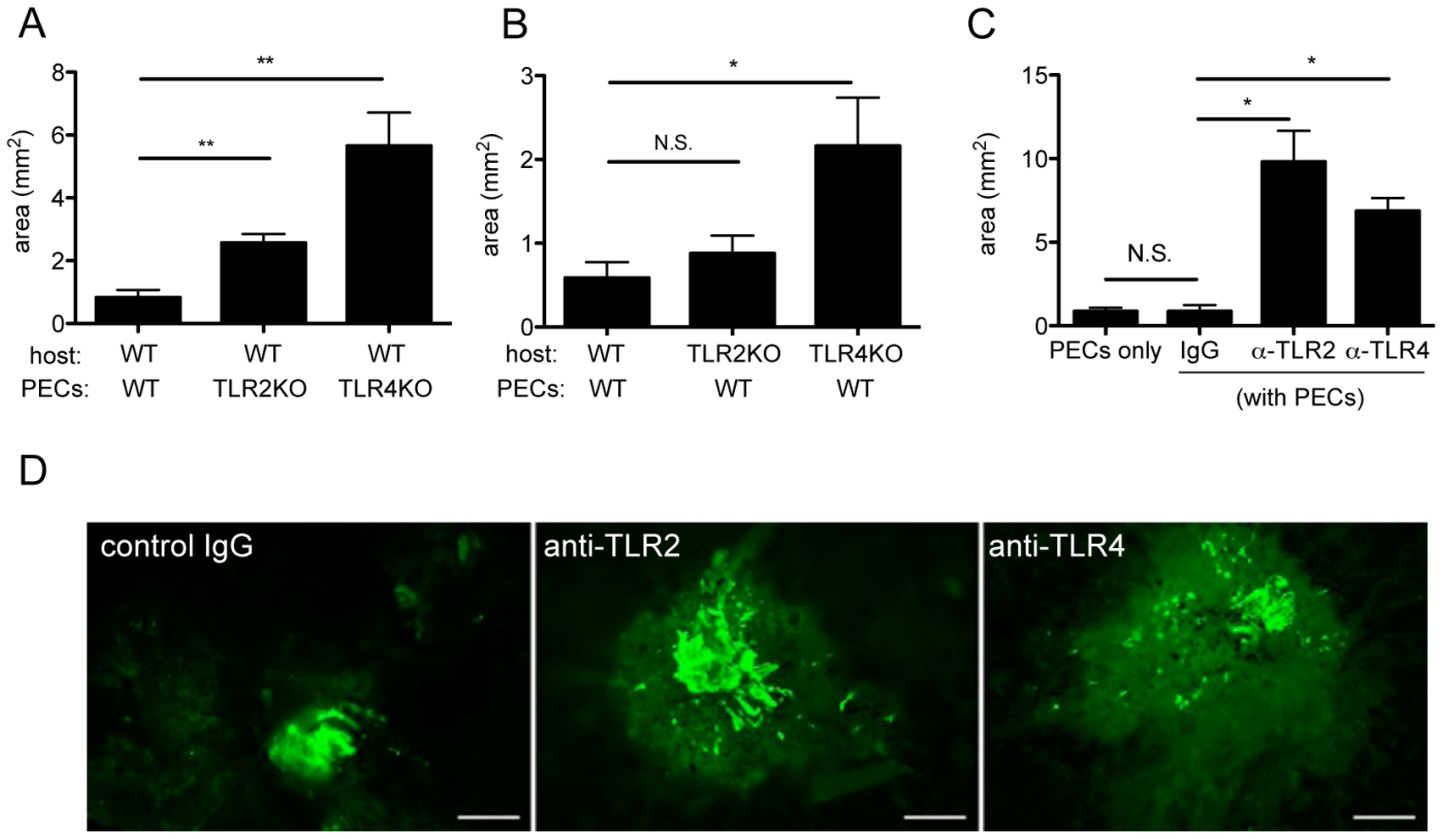
Both TLR2 and TLR4 signaling pathways are significantly involved in the formation of subretinal fibrosis. Subretinal fibrotic models were generated as previously described [11]. Briefly, laser photocoagulation (wave length 532 nm, 0.1 s, spot size 75 um, power 200 mW) was performed to the retina to make subretinal bubble and rupture Bruch membrane in WT mice. PECs (4×10^4^) from indicated mice collected as Materials and Methods without any stimulation were inoculated into the subretinal space of indicated mice. (A) PECs from each WT, TLR2KO and TLR4KO mice were inoculated into subretinal space of WT mice (n = 5). (B) PECs from WT mice were inoculated into subretinal space of WT, TLR2KO and TLR4KO mice. (n = 5) (C) With neutralizing antibody anti-TLR2, anti-TLR4 antibody, control IgG or without any reagent (naïve), PECs from WT mice were inoculated into subretinal space of WT mice. After 7 days, eyes were enucleated, and choroidal flatmounts were prepared and stained with anti-GFAP antibody. The areas of subretinal fibrosis were measured by ImageJ. *p<0.05 versus control; double asterisks, **p<0.005 versus control. (n = 5) Data represents mean ± SEM. (D) Histological cross sections from (C) were stained with anti-GFAP antibody. Representative images were shown. Scale bars, 500 µm. Results are represents as mean ± SEM.

Subretinal fibrosis is a common outcome after subretinal hemorrhage in advanced age-related macular degeneration. Several studies suggested that both TLR2 and TLR4 are implicated in the formation of fibrosis [3], [4], [13], [14]. These observation prompted us to examine the role of TLR2 and TLR4 signaling in subretinal formation. PECs from WT, TLR2KO and TLR4KO were inoculated into subretinal space of WT mice as described in *Materials and Methods* section. 7 days after PEC inoculation, eyes were enucleated and choroidal flatmount were prepared. Then the area of subretinal fibrosis was evaluated by staining with FITC-conjugated GFAP antibody. As shown in Figure 1A, the area of subretinal fibrosis was exacerbated by PECs in both TLR2KO and TLR4KO mice compared to WT C57BL/6 mice. In the previous report, we show that endogenous macrophages are recruited to form focal fibrosis with inoculated exogenous macrophages [11]. To analyze the environmental effects of TLR2 and TLR4 signaling in response to inoculated PECs, PECs from WT were inoculated in WT, TLR2KO and TLR4KO mice. As shown in Figure 1B, the exacerbated area of subretinal fibrosis was revealed in TLR4KO mice, whereas comparable area in TLR2KO mice. To further show the effect of TLR2 and TLR4 signaling in experimental subretinal fibrosis, a neutralizing antibody against TLR2 (1 ng/ml, 0.25 µl), TLR4 (1 ng/ml, 0.25 µl) with WT mice, or isotype IgG (1 ng/ml, 0.25 µl) was mixed with PECs, then inoculated into subretinal space. As shown in Figure 1C, a significant increase of fibrosis area was seen in both groups with the neutralizing antibody against TLR2 and TLR4, compared with the control group (Figure 1C and Figure 1D). These data clearly show that TLR2 and TLR4 signaling plays a significant role in subretinal fibrosis formation.

### Amelioration of Subretinal Fibrosis by HSP70 via TLR2 and TLR4

**Figure 2:**
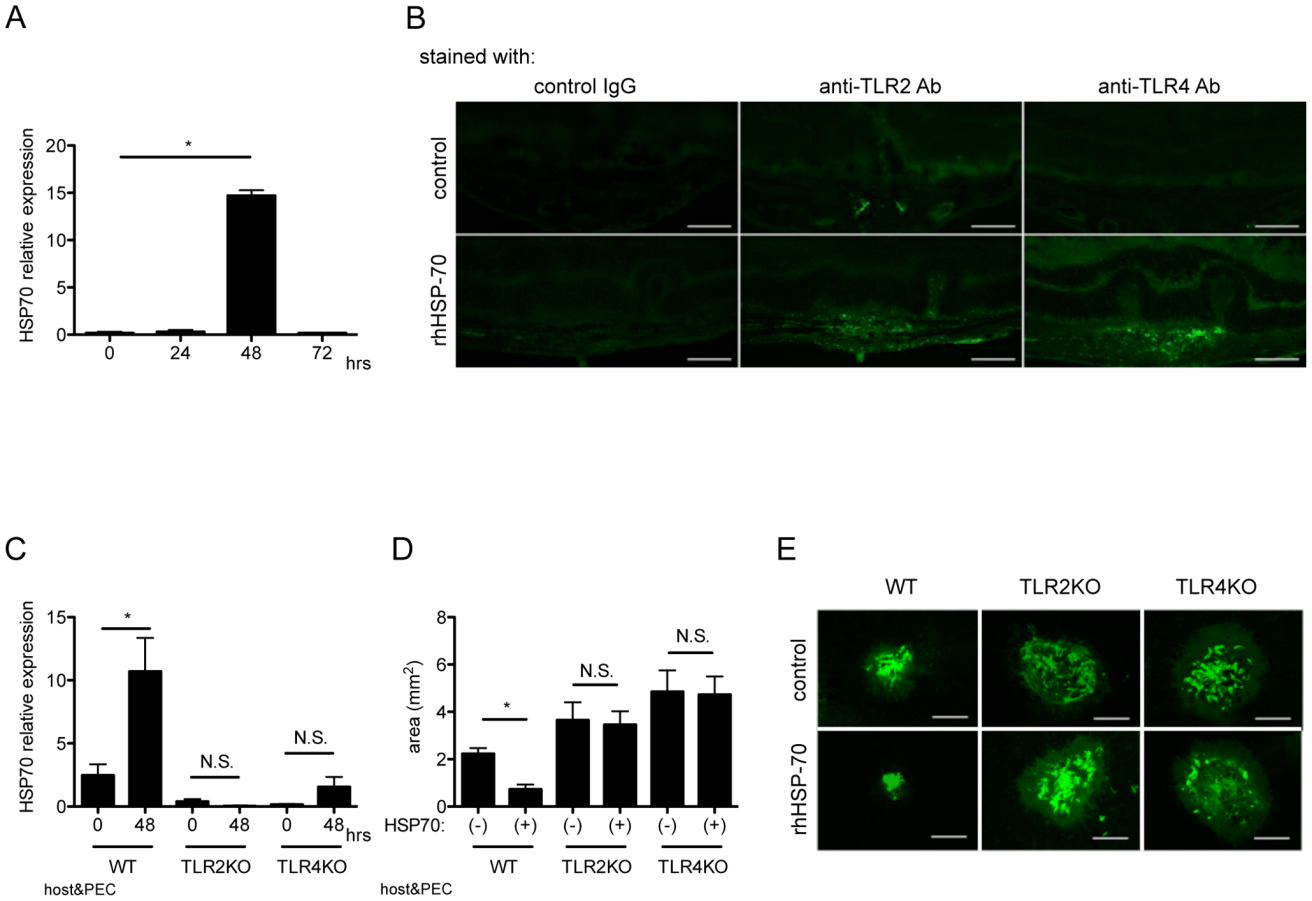
Role of HSP70 in the formation of subretinal fibrosis. (A) 0, 24, 48 and 72 hours after PEC inoculation, total RNA was extracted from the eyes and the amounts of HSP70 mRNA were assessed by quantitative real-time RT-PCR. (B) 72 hours after PEC inoculation following intravitreal HSP70 injection, eyes were enucleated, then histological cross sections at the site of subretinal fibrosis were stained with control IgG, anti-TLR2 and anti-TLR4 antibody, respectively. Representative images were shown. Scale bars, 100 µm. (C) Indicated hours after PECs inoculation, total RNA was extracted from eyes of each WT (WT PEC into lasered WT mice), TLR2KO (TLR2KO PEC into lasered TLR2KO) and TLR4KO (TLR4KO PEC into lasered TLR4KO) mice. The amount of HSP70 mRNA were evaluated by quantitative real-time PCR (n = 9). (D) Recombinant human HSP70 or control PBS was injected into vitreous cavity of WT, TLR2KO and TLR4KO mice 2 hours after PEC inoculation. After 7 days, eyes were enucleated, and choroidal flatmounts were prepared and stained with anti-GFAP antibody. The area of subretinal fibrosis were measured by ImageJ. (n = 5) (E) Representative images of choroidal flatmount stained with anti-GFAP antibody. Scale bars, 500 µm. Results are represents as mean ± SEM.

Heat shock proteins have been known to protect against various stressors and have anti-inflammatory activity, which can be mediated through both TLR2 and TLR4 activation [6], [15]. To examine the possible immunoregulatory role of HSP70, the kinetics of HSP70 expression during subretinal fibrosis were examined. 0, 24, 48 and 72 hours after WT PEC inoculation into subretinal space of WT mice, mRNA from eyes were extracted, and were subjected to quantitative real-time PCR. As shown in Figure 2A, HSP70 mRNA expression was significantly elevated 48 h after PEC inoculation. Whereas IL-6 was only detected at day 2, IL-10 was increased as day 2 and reduced at day 5 and 7. HSP70 levels increased at days 2, 3 and 5, and returned back to basal levels at day 7 after PEC inoculation (Figure S1). To confirm TLR2 and TLR4 activation in response to HSP70 in vivo, recombinant HSP-70 (25 ng/ml, 2 µl) was injected into vitreous cavity at the time of WT PEC inoculation. 72 hours after PEC inoculation following intravitreal HSP70 injection, eyes were enucleated, then histological cross sections at the site of subretinal fibrosis were stained with control IgG, anti-TLR2 and anti-TLR4 antibody, respectively. As shown in Figure 2B, HSP70 protein itself have revealed to be express both TLR2 and TLR4 in retinal tissue (Figure 2B). To further examine the role of HSP70 via activation of TLR2 and TLR4, we took advantage to use TLR2KO and TLR4KO mice. As shown in Figure 2C, HSP70 expression at 48 h after PEC inoculation was significantly lower in eyes from both TLR2 and TLR4KO mice compared with WT mice. To further examine the role of HSP70 in the formation of subretinal fibrosis, exogenous recombinant HSP70 (25 ng/ml, 2 µl) was injected into vitreous cavity at the time of PEC inoculation. As shown in Figure 2D, the area of subretinal fibrosis was significantly lower in the HSP70-injected group, as compared with control group. Following HSP70 intravitreal injection, however, no significant changes were observed in both TLR2 and TLR4 mice (Figure 2D, E). Taken together, these results show that HSP70 plays a significant role in ameliorating subretinal fibrosis formation through activation of TLR2 and TLR4.

### Hsp70 Induces Intraocular Production of IL-10 in Response to PEC Inoculation

**Figure 3:**
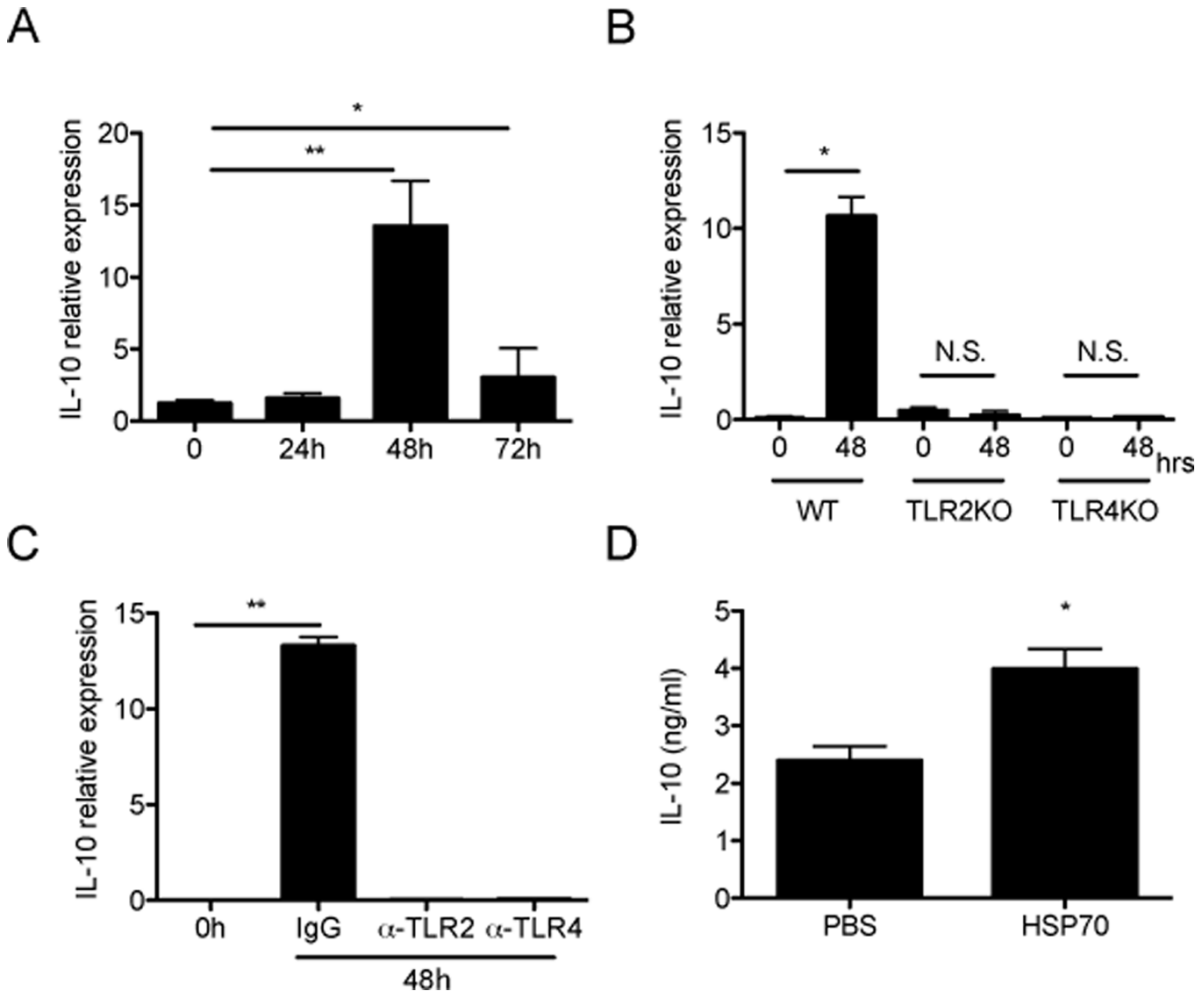
Implications of IL-10 and its possible association of HSP70 in the formation of subretinal fibrosis. (A) PECs from WT mice were inoculated into the subretinal space of WT mice. 0, 24, 72 hours after, total RNA was extracted from the eyes and the amounts of IL-10 mRNA were assessed by quantitative real-time RT-PCR. (n = 6) (B) PECs from WT were inoculated to each WT, TLR2 and TLR4 mice. 48 hours after PEC inoculation, total RNA was extracted from the eyes of each mice and the amount of IL-10 mRNA was assessed by quantitative real-time RT-PCR. (n = 6) (C) 2 hours after PEC inoculation, each control IgG, anti-TLR2 and anti-TLR4 neutralizing antibody was injected into vitreous cavity of WT mice. After 48 hours, total RNA was extracted from the eyes and the amounts of IL-10 mRNA were asessed by quantitative real-time RT-PCR. (n = 5) (D) 2 hours after PEC inoculation into subretinal space of WT mice, recombinant human HSP70 or control PBS was injected into vitreous cavity. After 48 hours, eyes were enucleated, total protein was extracted from the retina-choroid-RPEs and amounts of IL-10 were quantified by ELISA (n = 12). Results are represents as mean ± SEM.

Although the anti-inflammatory property of heat shock proteins (HSP) has been demonstrated in various animal models of inflammatory diseases [7], [16], [17], the mechanisms underlying these are not fully understood. A potential mechanism could be mediated by induction of anti-inflammatory cytokine IL-10 since the significance of IL-10 in dampening the inflammation is extensively described [8], [18]. To examine the possible role of IL-10 in the formation of subretinal fibrosis, the kinetics of intraocular production of IL-10 after PEC inoculation were examined. 0, 24, 48 and 72 hours after PEC inoculation, total mRNAs were extracted from each eye, subjected to quantitative real-time PCR. As shown in Figure 3A, intraocular IL-10 expression was greatly upregulated at 48 h after PEC inoculation. Both TLR2 and TLR4KO failed to induce intraocular IL-10 expression at 48 h after PEC inoculation (Figure 3B). Moreover, the neutralizing antibody against both TLR2 and TLR4 failed to induce intraocular IL-10 production (Figure 3C). Protein levels of IL-10 were detected from eyes at 72 h after PEC inoculation that had received exogenous HSP70 (Figure 3D). These results demonstrate that both TLR2 and TLR4 signaling have significant role in the production of intraocular IL-10 in response to PEC inoculation to subretinal space.

### IL-10 is Responsible for Suppressing Subretinal Fibrosis Formation

**Figure 4:**
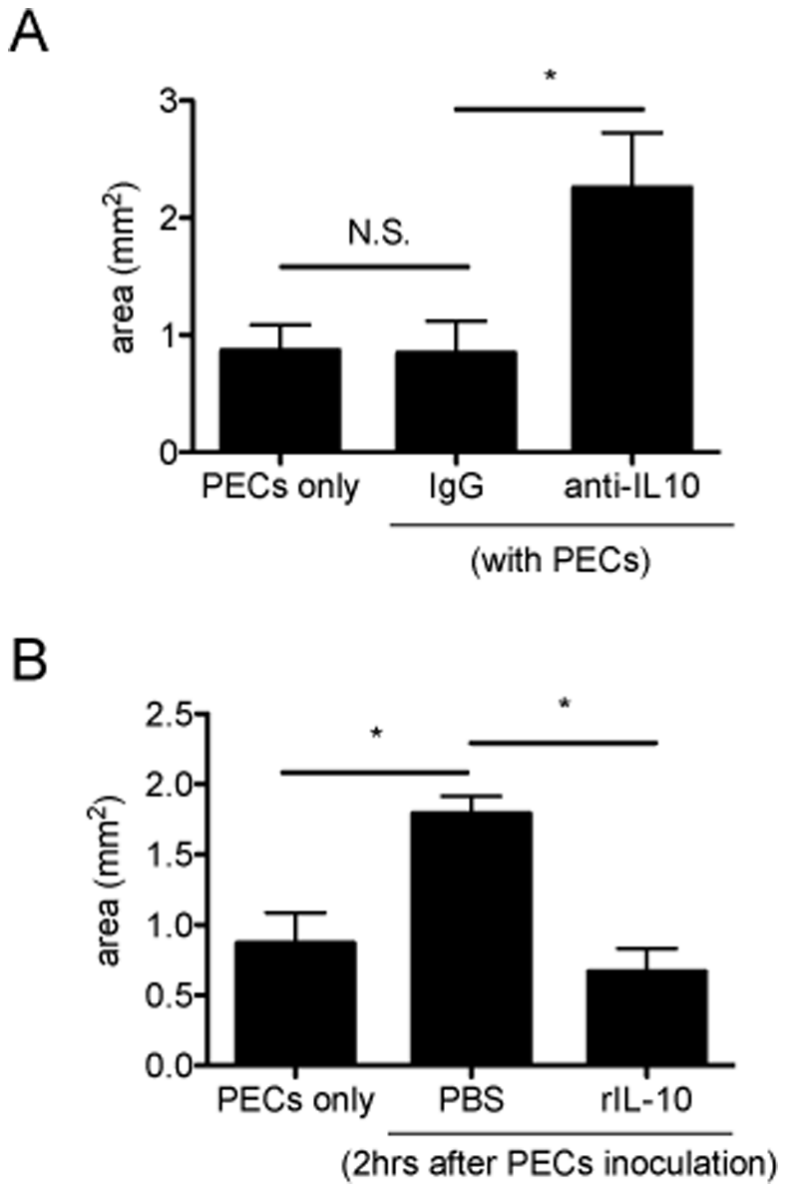
IL-10 is responsible for subretinal formation. (A) Neutralizing anti-IL-10 antibodies were inoculated with PECs from WT to subretinal space of WT mice. (B) 2 hours after PEC inoculation, recombinant IL-10 was intravitreally injected. After 7 days, eyes were enucleated, and choroidal flatmounts were prepared and stained with anti-GFAP antibody. The areas of subretinal fibrosis were measured by ImageJ. Results are represents as mean ± SEM.

Next, the direct effects of IL-10 on subretinal fibrosis were evaluated. A neutralizing antibody against IL-10 (1 µg/ml, 0.25 µl) or isotype control IgG was mixed with PEC and inoculated into subretinal space. The area of fibrosis was then assessed by immunohistochemistry. As shown in Figure 4A, the area of the group that was injected with the TLR2 and TLR4 neutralizing antibody was significantly larger compared with the control group. Next, recombinant IL-10 (0.6 ng/ml, 2 µl) or control PBS was administered intravitreally 2 hours after PEC inoculation. The area of subretinal fibrosis in the group that received IL-10 was significantly suppressed compared with the control group (Figure 4B). These results clearly demonstrated that IL-10 alone could play significant role in suppressing subretinal fibrosis formation.

### HSP70 Augmented IL-10 Production by RPE Cells via TLR2 and TLR4 Activation

**Figure 5:**
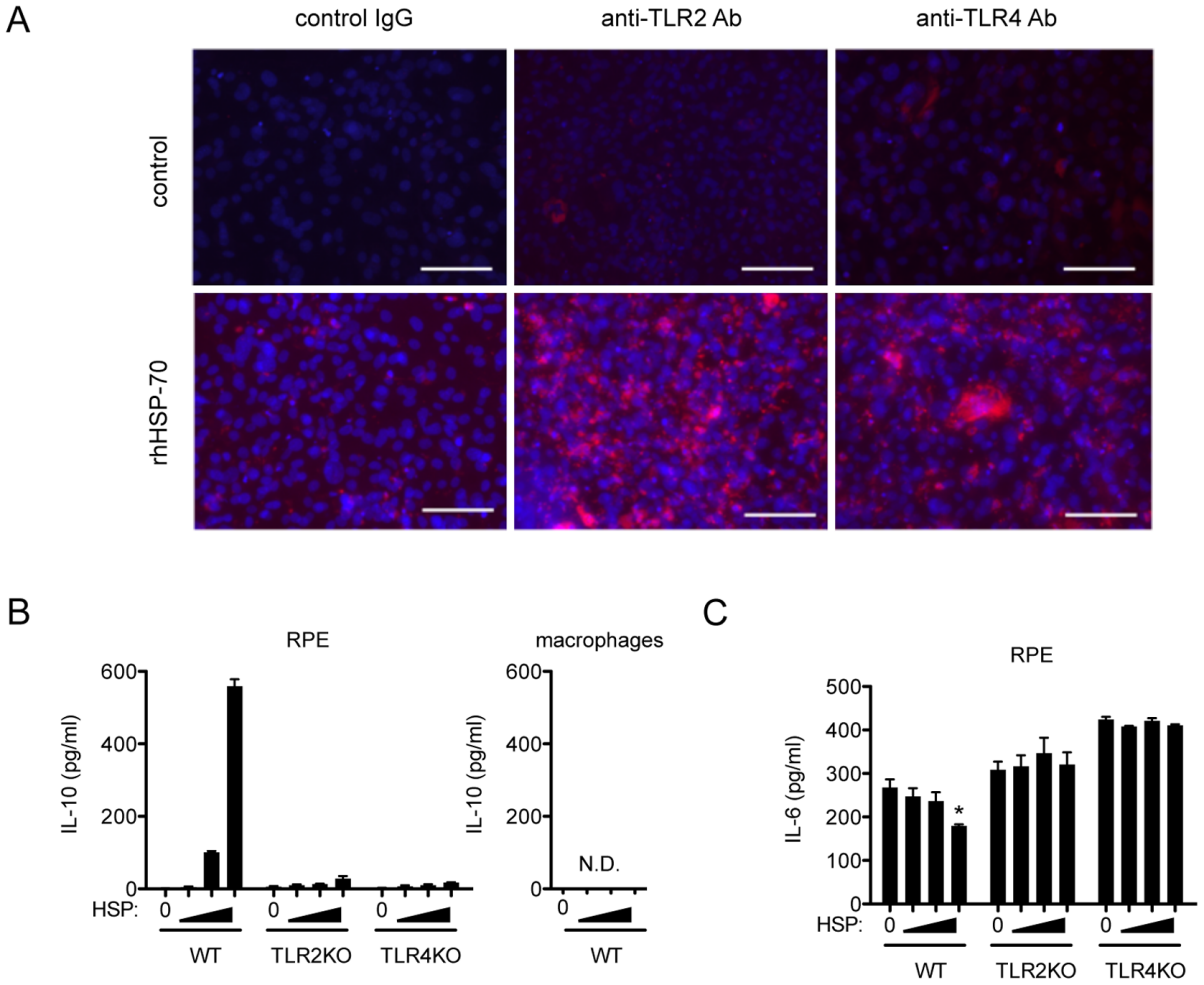
HSP70 induces IL-10 production by cultured RPEs but not by macrophages. (A) Cultured RPE cells in the presence or absence of rhHSP70 were stained with WT, anti-TLR2 and anti-TLR4 antibody, respectively. Representative images were shown. Scale bars, 100 µm. (B) RPE cells from each WT, TLR2 and TLR4 (*left*) and macrophages (*right*) were stimulated with 0.1, 0.3 and 1 ng/ml of recombinant human Hsp70 for 48hours, then supernatants were harvested and the levels of IL-10 were quantified by ELISA. (C) IL-6 was quantified by ELISA from same supernatants from (Figure B, *right*) RPE cells. Each experiment was representative of at least two experiments with similar results. Results are represents as mean ± SEM.

Retinal pigment cells produce IL-10 and are responsible for intraocular immunosuppressive mechanism [9], [19]. To examine whether cultured RPEs can augment IL-10 in response to HSP70, RPE cells were stimulated in the presence or absence of recombinant HSP70 for 48 hours. The expression of both TLR2 and TLR4 were clearly detected by immunohistochemistry (Figure 5A). Culture supernatants were collected, then subjected to ELISA. As shown in Figure 5B, exogenous HSP70 augmented IL-10 production from cultured RPE cells from WT mice in a dose-dependent manner, whereas RPE cells from both TLR2 and TLR4 did not produce any detectable IL-10. We then postulated that macrophages would produce IL-10 by HSP70 stimulation as well. However, cultured macrophages failed to produce IL-10 in response to HSP70; instead, the blockade of proinflammatory cytokine IL-6 modulates experimental subretinal formation (manuscript in preparation). We therefore postulated that HSP70 would suppress IL-6 production. As shown in Figure 5C, a high dose (1 ng/ml) of HSP70 suppressed IL-6 production, whereas RPEs from both TLR2 and TLR4 deficient mice produced comparable levels of IL-6 in response to HSP70 stimulation.

### Contamination of LPS Does Not Affect the Immunomodulatory Role of HSP70

**Figure 6:**
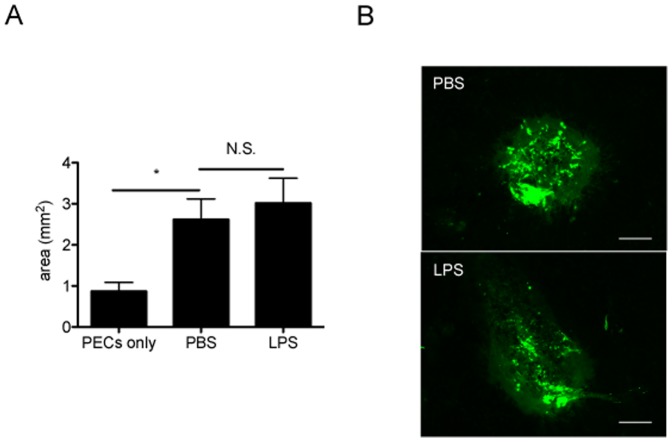
The effects of human Hsp70 on IL-10 production in subretinal fibrotic model are not due to the contamination of LPS. (A) 2 hours after PECs-inoculation, 50 ng (25 µg/ml, 2 µl) of lipopolysaccharides (LPS) or PBS was administered into vitreous cavity in WT mice. After 7 days, eyes were enucleated, choroidal flatmounts were prepared and stained with anti-GFAP antibody. The areas of subretinal fibrosis were measured by ImageJ. Results are represents as mean ± SEM. (B) Histological cross sections from (A) were stained with anti-GFAP antibody. Representative images were shown. Scale bars, 100 µm.

Recent studies have shown that contamination of Hsp70 with LPS might be responsible for its stimulatory activation on macrophages and dendritic cells [20]. To exclude this possibility, we first confirmed that recombinant human Hsp70 used in this study contained <0.05 EU/µg protein (5 pg/µg) of bacterial endotoxin. Then an equal amount of LPS (25 µg/ml, 2 µl) was intravitreally injected following PEC inoculation. According to previous studies [21], it was lower than the dose which would cause uveitis. As shown in Figure 6, the size of subretinal fibrotic area was comparable between control PBS and LPS-injected eyes. These data clearly confirmed that LPS contamination in the recombinant HSP70 protein was minimal.

### Possible Direct Effect on HSP70 and IL-10 in the Formation of Subretinal Fibrosis

**Figure 7:**
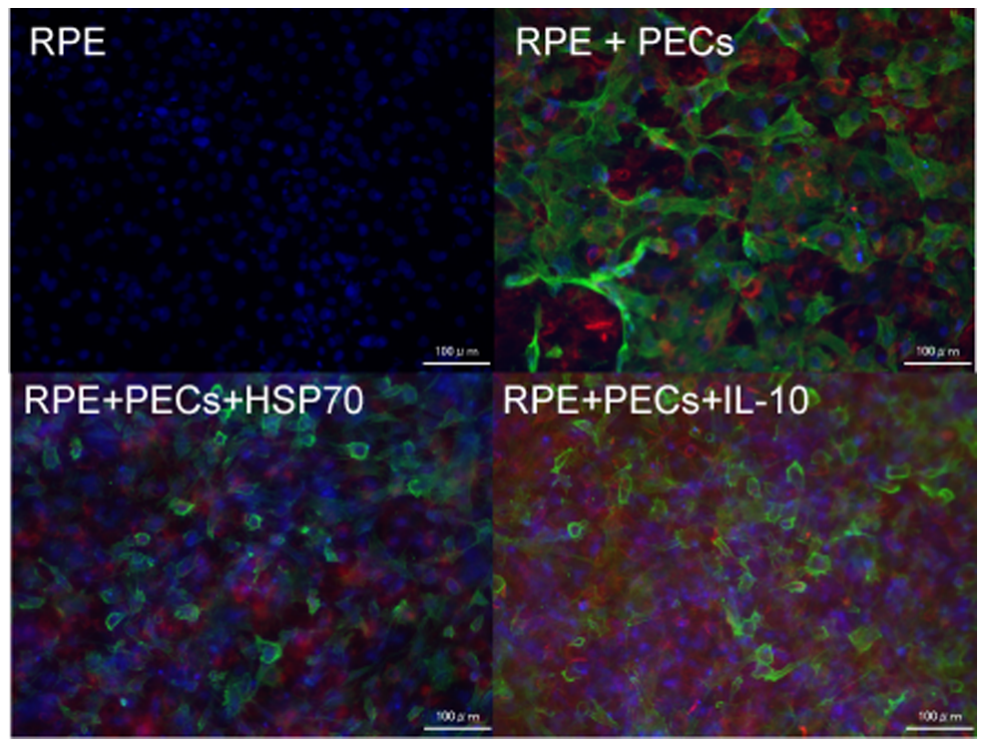
Suppressive effect of HSP70 and IL-10 on α-SMA expression in the RPE cells cultured with PECs. RPE cells were prepared from C57BL/6 mice and cultured approximately 2 weeks until becoming confluent in a 24 well plate. PECs were added to the primary culture in the absence or presence of HSP70 (1 ng/ml) or IL-10 (10 ng/ml). 48 hours later, cells were stained by FITC-conjugated α-SMA and PE-conjugated anti-F4/80 antibody at 4°C for 24 hours. All samples were counterstained with DAPI, mounted, and subjected to fluorescence microscopy. Representative images of α-SMA-stained RPE cells (green) and F4/80-stained macrophages (red) on the dish were shown.

In this model, the formation of fibrosis relays on laser-induced CNV formation. IL-10 has been shown to be anti-angiogenic in the mouse model of CNV [22]. The reduction in fibrosis in TLR2/4 KO and in HSP70/IL-10 treated mice could be the indirect results from the reduced CNV formation, but not the direct effect of the treatment on fibrosis. This possibility attempted us to assess direct effect of HSP70 and IL-10 in the formation of subretinal fibrosis. PECs were added to primary cultured RPE cells from C57BL/6 in the absence or presence of HSP70 or IL-10. 48hours later, cells were stained with FITC-conjugated α-SMA and anti-F4/80. As shown in Figure 7, each HSP70 and IL-10 treatment clearly suppressed α-SMA expression on RPE cells. Intracellular flow cytometry analysis revealed that HSP70 treatment suppresses intracellular expression of α-SMA (data not shown) on RPE cells. These data implicated the direct suppressive effect of HSP70/IL-10 on experimental subretinal formation.

## Discussion

Substantial progress has been made towards the elucidation of the pathophysiology of angiogenesis and the role of anti-angiogenic therapies in the treatment of pathophysiological neovascularization [23]. To date, inhibition of angiogenesis is one of the leading therapeutic approaches in neovascular diseases such as diabetic retinopathy and AMD. However, focal subretinal fibrosis following the infiltration of exudative leukocytes would be an important cause of severe visual loss. In this study, we show the importance of HSP70 in subretinal formation by inducing immunomodulatory cytokine IL-10. Blockade TLR2 and TLR4 resulted in exacerbating subretinal fibrosis.

The anti-inflammatory properties of HSPs have been shown in several studies in both animal models and in patients suffering from inflammatory diseases [16]. However, for further development of HSPs for therapeutic application, it is essential to understand the mechanisms by which HSPs affect inflammatory disease in more detail. In this study we analyzed the effect of HSP70 on focal subretinal fibrosis. Our data clearly show that injection of exogenous HSP70 dramatically reduced the area of subretinal fibrosis. In TLR2- and TLR4-deficient mice, exogenous HSP70 administration fail to suppress fibrosis, illustrating the TLR2- and TLR4-dependent mechanism of HSP70 induced immunoregulation. In addition, in vitro data show that extracellular HSP70 can induce anti-inflammatory cytokine IL-10 following the inoculation of PECs into subretinal space possibly through autocrine or paracrine stimulation of TLR2 and TLR4-mediated interaction.

Although HSPs were once regarded as intracellular proteins, there is growing evidence suggesting that HSPs can also be expressed on cellular membranes and even released into the extracellular environment after stress. HSPs have been found in the circulation of both healthy individuals and those suffering from autoimmune diseases and inflammatory conditions [10] [24]. In earlier studies, it was suggested that cellular Hsp70 release may be the result of cell lysis, but it is now recognized that elevated Hsp70 may be found in the absence of necrosis. In fact, Hsp70 could be actively released from glial cells [25], epithelial cells [26], tumor cells [27], PBMC, [28] and importantly, human eyes [29].

Extracellular HSPs have previously been thought of as initiators of a pro-inflammatory response within the innate immune system. It has been shown that extracellular Hsp70 and Hsp60 can induce the production of pro-inflammatory cytokines including TNF-α, IL-1, and IL-6 in monocytes and macrophages [30] [31]. Recently, there is evidence that, in contrast to the pro-inflammatory response, some extracellular HSPs, including human Hsp60 [32], human Hsp27 [33], and human Hsp10, [34] induce a strong anti-inflammatory response with sustained production of IL-10 in vitro and in vivo. Human Hsp60 treatment of T cells in vitro was found to inhibit the production of pro-inflammatory cytokine TNF-α and IFN-γ, and trigger the production of anti-inflammatory cytokines IL-10 [32]. TGFβ stimulation considered to cause much higher levels of IL-10 secretion, however, pretreatment of TGFβ did not alter subretinal fibrosis formation (Figure S2). Although further study needed to clarify the precise mechanism of intraocular inflammatory balance to form subretinal formation, these findings suggest that, rather than being proinflammatory, self HSP reactivity might be a physiological mechanism for regulation of pro-inflammatory responses and inflammatory disease. It should therefore not be surprising if self Hsp70 is found to have an anti-inflammatory effect in this model of subretinal fibrosis. Indeed, our studies showed that human Hsp70 significantly induced the production of anti-inflammatory cytokine IL-10 but failed to induce the production of pro-inflammatory cytokines including IL-6 in RPE cells (Figure 5), suggesting that extracellular Hsp70 have an anti-inflammatory property in the formation of subretinal fibrosis. Thus, it is conceivable that in subretinal fibrosis which is due to CNV, inflammatory stress contributes to the expression and release of Hsp70 and the extracellular Hsp70 may act as natural dimmers of inflammation by inducing RPEs to produce IL-10, which is a part of the normal mechanism to down-regulate an inflammatory response.

The TLRs are a family of pattern recognition receptors, through which cells of the innate immune system, including macrophages and dendritic cells, recognize microbial pathogens [35]. So far, ten different proteins have been defined as members of the human TLR family. Pathogen-associated molecular patterns such as LPS are recognized by TLR4 and TLR2. TLRs have been shown to mediate activation of NF-κB and mitogen-activated protein kinase signaling pathways, resulting in the production of pro-inflammatory mediators such as IL-1, IL-6, IL-8, or TNF-α from the innate immune cells [35] [36], [37]. Hsp70 has been reported to be recognized by TLR2 and TLR4 [9], which are abundantly expressed on innate immune cells such as macrophages and dendritic cells [38]. Zhou et al. reported that heat shock upregulates expression of TLR2 and TLR4 in human monocytes via p38 kinase signal pathway [39]. Although several studies have demonstrated that TLR2 and TLR4 are also expressed in a variety of non-immune cells, their function is less well understood. Our results demonstrated that fibrotic areas increased without TLR2 and TLR4 signaling (Figure 1), which in turn suggests that Hsp70 might affect the formation of subretinal fibrosis by its binding to TLR2 and TLR4. As we have shown here, TLR2 and TLR4 deficient mice and a specific monoclonal antibody (mAb) to TLR2 and TLR4 suppressed the production of intraocular IL-10 after PEC inoculation, indicating that the effect of Hsp70 on fibrosis would depend on TLR2 and TLR4 signaling pathways in the IL-10 production.

There are limitations of this study. Kelly et al. [40] demonstrated that IL-10 augment laser-induced CNV size. In this present study, IL-10 suppressed the formation of subretinal fibrosis, but this could be due to reduced CNV but not fibrosis per se. As described in elsewhere [41], M1 macrophage secrete TNF-α, IL-1, IL-6, and be implicated in the formation of choroidal neovascularization. Kleinman et al. [42] described the role of proinflammatory cytokine IL-12 and IFN-γ as anti-angiogenic agent. IL-10 has been reported to be accelerating CNV [43]. There are still numerous discussions whether proinflammatory or anti-inflammatory cytokine has pro-angiogenic or anti-angiogenic nature. As we have previously reported [11], not only exogenous macrophages but also intrinsic macrophages are activated to form subretinal fibrosis. Although we provided the possible evidence that exogenous HSP and IL-10 directly suppress the fibrosis formation in vitro (Figure 7), further study needed to clarify this point.

Other experimental limitations should be discussed. For example, as shown in Figure 1C, 2D, 4A and 4B, the subretinal lesion size in different control groups varies significantly in different studies. These may be due to the difference of experimental settings in each group; to be much concentrated in subretinal space, neutralizing antibody was mixed with PEC suspension and then inoculated into subretinal space (single injection). However, not worked well with same protocol above, recombinant proteins were intravitreally injected 2 hours after PEC injection (twice injection, Figure 4B and 6). The latter surgical setting might reflect undefined inflammation which may alter subretinal lesion size, resulted in varied basal levels. Moreover, Figure 3A shows that IL-10 mRNA was upregulated at 72 h, whereas in Figure S1 it shows that IL-10 protein levels was normal at 72 h and lower at day5 (than day 0). At day 0 in Figure 3A and Figure S1, eyes were enucleated just after PECs inoculation, which detect IL-10 from combination of exogenous PECs and naive eye. Each base line IL-10 mRNA and protein level at Figure 3A and Figure S1, respectively, is that from activated macrophages and naïve eyes, then mRNA and transcript protein levels alter as inflammation proceed. In Figure S1 we extracted protein from retina-RPE-choroid complex that contains activated macrophages; it could lead to overdetection of both temporary released IL-10 and already existed intracellular IL-10. Therefore it could be reasonable to detect lower levels of IL-10 protein in late phase (day 3 and 5). Overall, this experimental system still has room for improvement in the future.

**Figure 8:**
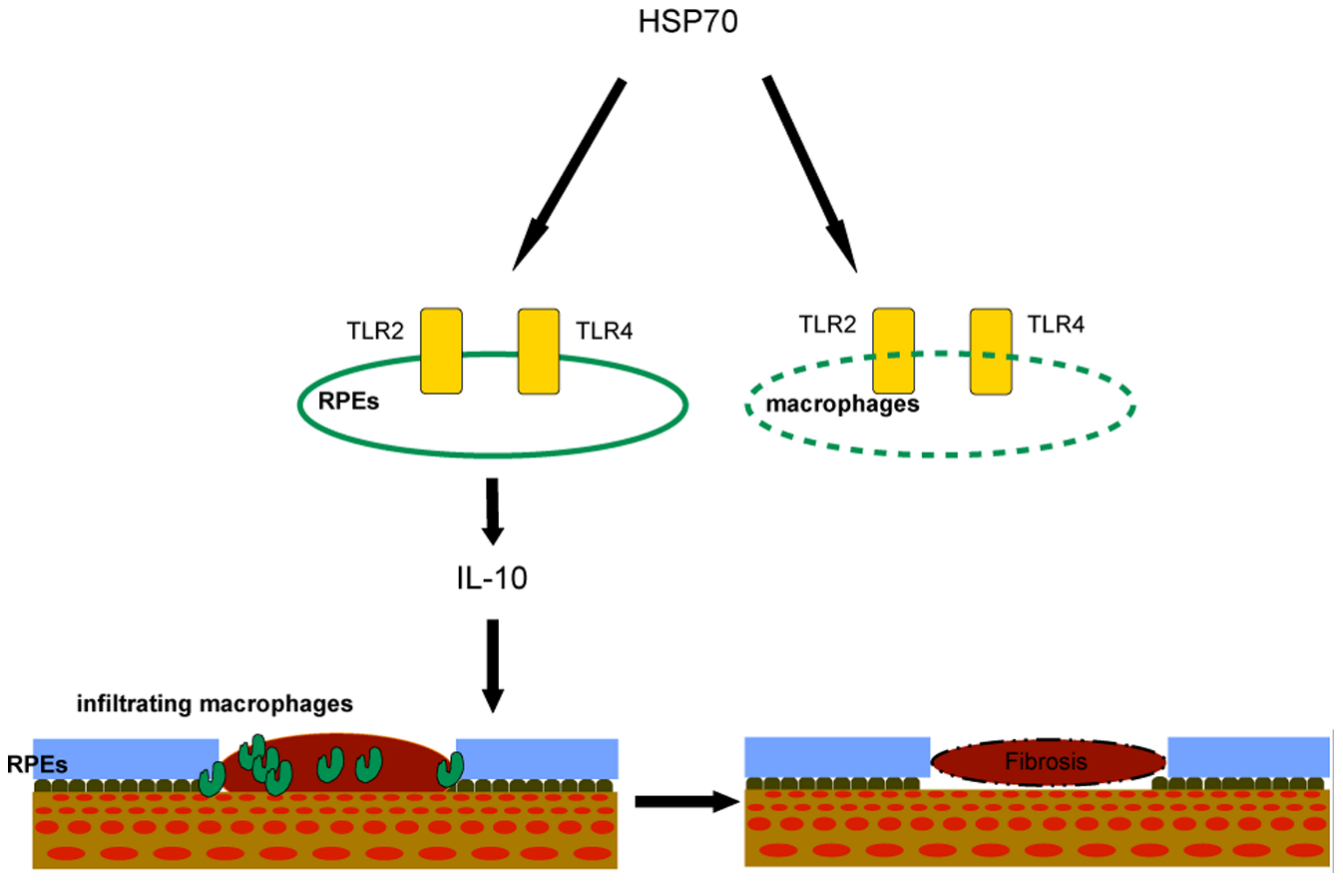
Possible mechanism of HSP70 in the regulation of subretinal formation. Intraocular expression of HSP70 activates TLR2 and TLR4 signaling cascade in RPEs but not in macrophages, results in the regulation of subretinal formation via production of IL-10.

In summary, we show that exogenous Hsp70 can be a major paracrine/autocrine inducer of IL-10 production in RPE cells via TLR2 and TLR4, resulted in reduced subretinal fibrosis (schema in Figure 8). The results of this study indicate that exogenous Hsp70 can be ideal candidates for immunotherapy against chronic inflammatory diseases, which can form subretinal fibrosis such as AMD.

## Supporting Information

Figure S1 Kinetics of intraocular IL-6, IL-10 and HSP70 expression after PEC inoculation. On day 0, 2, 3, 5, 7 after PEC inoculation, protein from retina-RPE-choroid complex (n = 5 each) were subjected to ELISA (IL-6, IL-10 and HSP70). Results are represents as mean ± SEM.
(TIF)

Figure S2 Pretreatment of exogenous macrophage with LPS and TGFβ do not alter subretinal fibrosis formation. (A) PECs were cultured with serum-free medium (complete medium except for FCS) that was supplemented with 0.1% BSA and with 0.2% insulin, transferrin serenium (ITS)+culture supplement (Collaborative Biochemical Products, Bedford, MA). Each cells were stimulated with LPS (1 µg/ml) or or ΤGFβ (2 ng/ml) for 24hours. Culture supernatants were subjected to ELISA (IL-6 and IL-10). (B) Collected, resuspended PECs were inoculated into subretinal space of WT mice (n = 5). After 7 days, eyes were enucleated, and choroidal flatmounts were prepared and stained with anti-GFAP antibody. The area of subretinal fibrosis were measured by ImageJ. (n = 5) (C) Representative images of choroidal flatmount stained with anti-GFAP antibody (bar = 100 µm). Results are represents as mean ± SEM.
(TIF)
