# Correction: IL-10 Is Significantly Involved in HSP70-Regulation of Experimental Subretinal Fibrosis

**DOI:** 10.1371/journal.pone.0091197

**Published:** 2014-03-17

**Authors:** The PLOS ONE Staff

A Funding statement is incorrectly omitted from the article. The Funding statement should read: "This work was supported by JSPS KAKENHI Grant Number 23689071 [grant-in-aid for Young Scientists (A) (A.T.)], Grant Number 24791859 [grant-in-aid for Young Scientists (B) (T.Y.)], Grant Number 10294943 [grants B (K-H.S.)], and Grant Number 23592573 [grants C (Y.O.)] from the Ministry of Education, Science, Sports and Culture, Japan."
