# Supplementary figures and images for: IL-10 Is Significantly Involved in HSP70-Regulation of Experimental Subretinal Fibrosis
Source: PLoS One. 2013 Dec 20;8(12):e80288. doi: 10.1371/journal.pone.0080288 (PMC3869650; doi:10.1371/journal.pone.0080288)

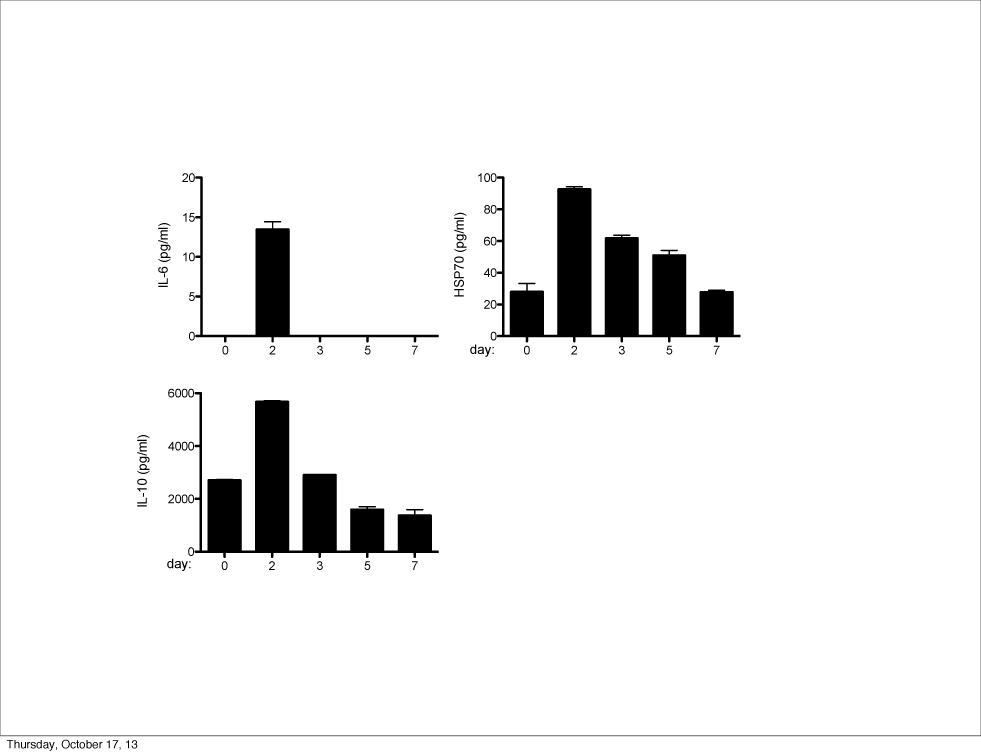

Supplement: Figure S1 — Kinetics of intraocular IL-6, IL-10 and HSP70 expression after PEC inoculation. On day 0, 2, 3, 5, 7 after PEC inoculation, protein from retina-RPE-choroid complex (n = 5 each) were subjected to ELISA (IL-6, IL-10 and HSP70). Results are represents as mean ± SEM. (TIF) [file pone.0080288.s001.tif]

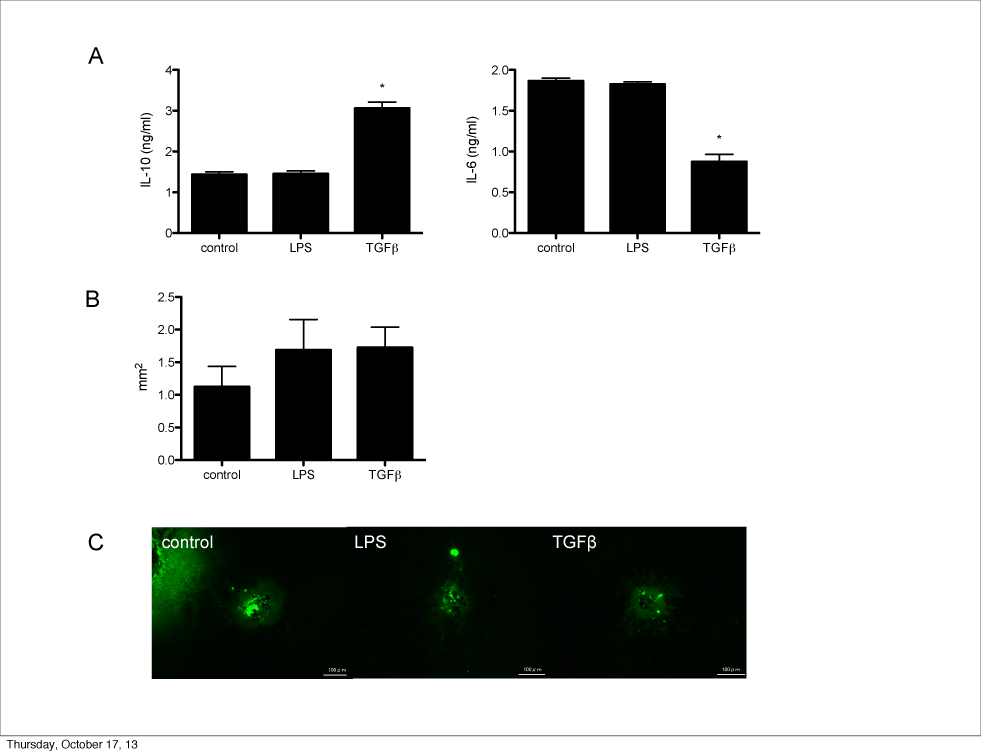

Supplement: Figure S2 — Pretreatment of exogenous macrophage with LPS and TGFβ do not alter subretinal fibrosis formation. (A) PECs were cultured with serum-free medium (complete medium except for FCS) that was supplemented with 0.1% BSA and with 0.2% insulin, transferrin serenium (ITS)+culture supplement (Collaborative Biochemical Products, Bedford, MA). Each cells were stimulated with LPS (1 µg/ml) or or ΤGFβ (2 ng/ml) for 24hours. Culture supernatants were subjected to ELISA (IL-6 and IL-10). (B) Collected, resuspended PECs were inoculated into subretinal space of WT mice (n = 5). After 7 days, eyes were enucleated, and choroidal flatmounts were prepared and stained with anti-GFAP antibody. The area of subretinal fibrosis were measured by ImageJ. (n = 5) (C) Representative images of choroidal flatmount stained with anti-GFAP antibody (bar = 100 µm). Results are represents as mean ± SEM. (TIF) [file pone.0080288.s002.tif]
